# Epigenomics and genotype-phenotype association analyses reveal conserved genetic architecture of complex traits in cattle and human

**DOI:** 10.1186/s12915-020-00792-6

**Published:** 2020-07-03

**Authors:** Shuli Liu, Ying Yu, Shengli Zhang, John B. Cole, Albert Tenesa, Ting Wang, Tara G. McDaneld, Li Ma, George E. Liu, Lingzhao Fang

**Affiliations:** 1 Animal Genomics and Improvement Laboratory, Agricultural Research Service, USDA, BARC-East, Beltsville, MD 20705 USA; 2 College of Animal Science and Technology, China Agricultural University, Beijing, 100193 China; 3 MRC Human Genetics Unit, Institute of Genetics and Molecular Medicine, University of Edinburgh, Edinburgh, EH4 2XU UK; 4 The Roslin Institute, University of Edinburgh, Edinburgh, EH25 9RG UK; 5 Department of Genetics, Washington University School of Medicine, St. Louis, MO 63110 USA; 6 US Meat Animal Research Center, Agricultural Research Service, USDA, Clay Center, NE 68933 USA; 7 Department of Animal and Avian Sciences, University of Maryland, College Park, MD 20742 USA

**Keywords:** Comparative epigenomics, GWAS enrichment, Trait-relevant tissues, Human-cattle comparison

## Abstract

**Background:**

Lack of comprehensive functional annotations across a wide range of tissues and cell types severely hinders the biological interpretations of phenotypic variation, adaptive evolution, and domestication in livestock. Here we used a combination of comparative epigenomics, genome-wide association study (GWAS), and selection signature analysis, to shed light on potential adaptive evolution in cattle.

**Results:**

We cross-mapped 8 histone marks of 1300 samples from human to cattle, covering 178 unique tissues/cell types. By uniformly analyzing 723 RNA-seq and 40 whole genome bisulfite sequencing (WGBS) datasets in cattle, we validated that cross-mapped histone marks captured tissue-specific expression and methylation, reflecting tissue-relevant biology. Through integrating cross-mapped tissue-specific histone marks with large-scale GWAS and selection signature results, we for the first time detected relevant tissues and cell types for 45 economically important traits and artificial selection in cattle. For instance, immune tissues are significantly associated with health and reproduction traits, multiple tissues for milk production and body conformation traits (reflecting their highly polygenic architecture), and thyroid for the different selection between beef and dairy cattle. Similarly, we detected relevant tissues for 58 complex traits and diseases in humans and observed that immune and fertility traits in humans significantly correlated with those in cattle in terms of relevant tissues, which facilitated the identification of causal genes for such traits. For instance, *PIK3CG*, a gene highly specifically expressed in mononuclear cells, was significantly associated with both age-at-menopause in human and daughter-still-birth in cattle. *ICAM*, a T cell-specific gene, was significantly associated with both allergic diseases in human and metritis in cattle.

**Conclusion:**

Collectively, our results highlighted that comparative epigenomics in conjunction with GWAS and selection signature analyses could provide biological insights into the phenotypic variation and adaptive evolution. Cattle may serve as a model for human complex traits, by providing additional information beyond laboratory model organisms, particularly when more novel phenotypes become available in the near future.

## Background

Understanding genetic and biological mechanisms underlying complex traits (e.g., causal variants and their corresponding tissues at specific physiological stages) is a main theme of research in the field of genetics and biology. In the past decade, genome-wide association studies (GWAS) have revealed thousands of genomic regions associated with a wide range of complex traits and diseases in human and other species [1–3]. However, the vast majority of GWAS hits locate outside protein-coding regions, hindering their biological interpretations and medical applications [4], while reflecting the important roles of regulatory regions in phenotypic diversity and adaptive evolution [5–7]. Therefore, researchers have put great efforts into the annotation of regulatory elements (e.g., promoters and enhancers) across multiple tissues and cell types in human and model organisms, such as ENCODE projects in human, mouse, and Drosophila [8–10] and human Roadmap Epigenomics Project [11]. By integrating such functional annotations with GWAS from large cohorts (e.g., UK biobank), investigators gained novel biological insights into the genetic architecture underlying complex traits and diseases in human [12–14]. For instance, Finucane et al. observed a significant association of central nervous system cells with body mass index and smoking behavior, through estimating the heritability enrichment of cell type-specific elements across multiple complex phenotypes [12]. Roadmap Epigenomics Consortium demonstrated that GWAS signals of late-onset Alzheimer’s disease were unexpectedly and significantly enriched in enhancers of immune cell types rather than brain tissues [11]. However, in livestock and other non-model organisms, lack of comprehensive functional annotations across multiple tissues and cell types severely limits our biological interpretations for their phenotypic diversity and adaptive evolution, although numerous genomic variants have been detected for thousands of complex phenotypes and positive selection in those animals [3]. The Functional Annotation of Animal Genomes consortium (FAANG), still in its early phase, aims to generate a comprehensive catalogue of regulatory elements for domestic and non-model organism species [15]. By constructing the first map of regulatory elements in the livestock species, we recently showed that GWAS signals of multiple complex traits were significantly enriched in active promoters and enhancers in bovine rumen epithelial primary cells [16].

The observation that epigenomes are generally conserved across species [17–21] facilitated the emergence of comparative epigenomics. This opened a new avenue to explore the biological basis of complex outcomes and adaptive evolution in the target species (e.g., cattle and swine) by borrowing functional annotations from well-studied species such as humans and mice. For example, Zhou et al. reported that the primary sequence conservation drove the conservation of tissue-specific DNA methylation among human, mouse, and rat [22]. Ebert et al. showed that predicted epigenomes by cross-species mapping could identify tissue-specific expression in the target species [23]. We thereby hypothesized that comparative epigenomics in conjunction with large-scale GWAS could enable us to explore the biological basis underlying complex traits of economic importance and positive selection in cattle. In addition, Holstein cattle has a unique population structure (e.g., strong selection and high inbreeding) and a large amount of phenotypic records measured with high accuracy [24, 25], including growth, health, and fertility traits. These may make Holstein cattle a potential animal model for studying certain human complex traits and diseases. Recently, by comparing sperm methylomes between human and Holstein cattle, we demonstrated that genomic variants of morphology-relevant traits were consistently and significantly enriched in the evolutionarily conserved hypomethylated regions in human and cattle [26]. Furthermore, the rapidly reduced immune and reproductive capacity of dairy cattle has been observed during the strong selection for milk production over the past decades [27]. Using these cattle resources, it is possible to extrapolate genomic changes associated with immune and reproduction in cattle to further advance human biomedical researches.

**Fig. 1:**
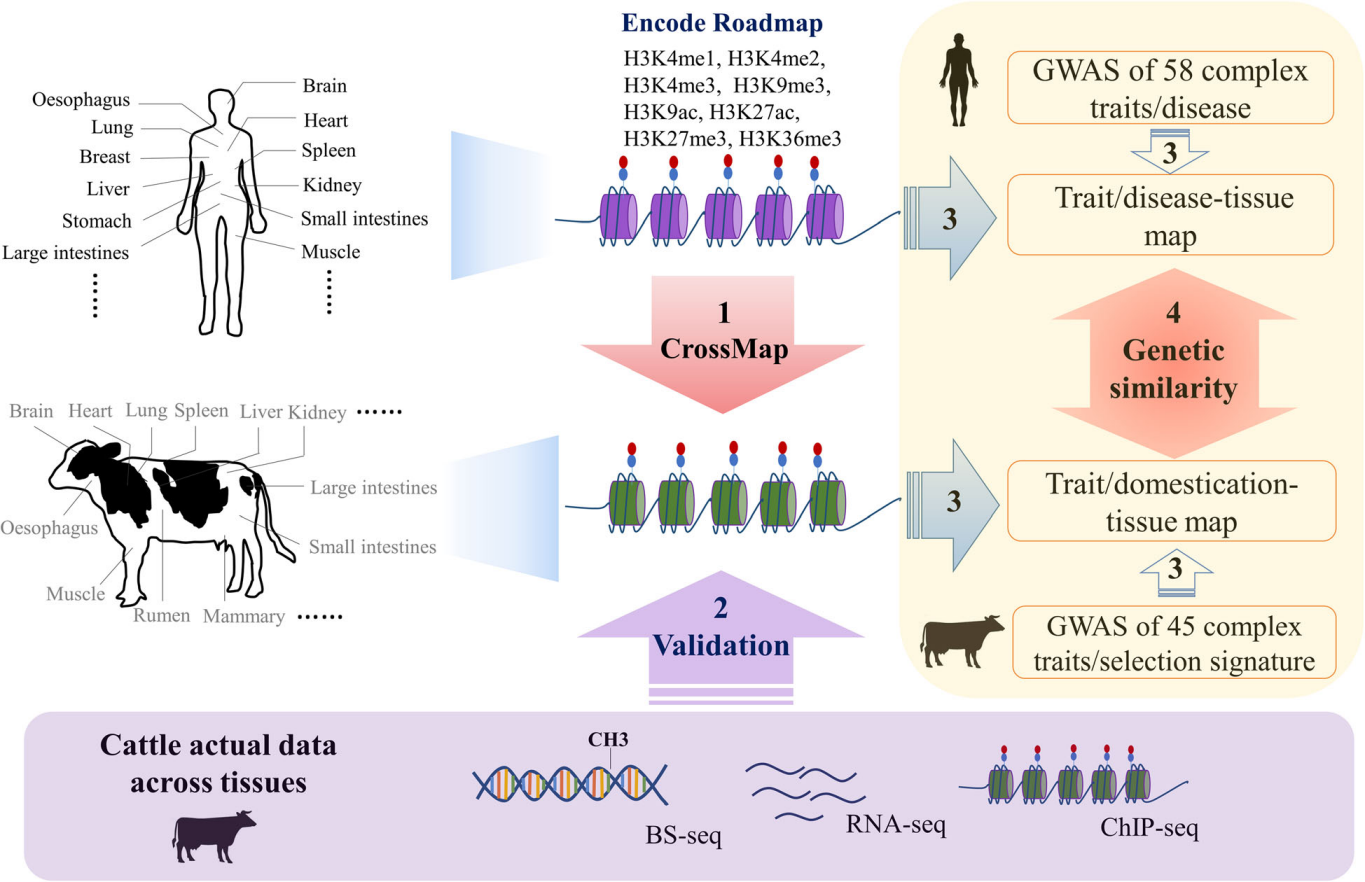
Schematic overview of current study. We retrieved human epigenome data, including eight histone marks (H3K4me1, H3K4me2, H3K4me3, H3K9me3, H3K9ac, H3K27ac, H3K27me3, and H3K36me3), from Encode and Roadmap, covering more than 100 tissues and cell types. We transferred the epigenome data from human to cattle using crossMap (1). We then validated the predicted epigenomes using measured ChIP-seq, 723 RNA-seq samples, and 40 whole genome bisulfite sequencing (WGBS) data in cattle (2). We detected relevant tissues for 45 complex traits and selection signature by integrating predicted tissue-specific histone marks with large-scale genome-wide association study (GWAS) in cattle. We further detected relevant tissues for the 58 matched complex traits/diseases in human (3) and explored the shared genetic architecture underlying complex traits between cattle and human (4)

In this study (Fig. 1), we first cross-mapped 1300 epigenomes (i.e., eight distinct histone marks) from human to cattle, including 178 tissues and cell types. We then validated cross-mapped epigenomes by analyzing experimentally generated ChIP-seq data of histone marks (*n* = 4), RNA-seq (*n* = 723) and whole genome bisulfite sequencing (WGBS; *n* = 40) in cattle. Through integrating cross-mapped epigenomes with 45 GWAS datasets (*n* = 27,214) and selection signature (Run 6 of the 1000 Bull Genome Project) in cattle [28], we for the first time identified relevant tissues or cell types for economically important traits and artificial selection in cattle. We further explored the shared genetic and biological basis of complex traits and diseases between cattle and human by similarly examining GWAS of 58 complex traits and diseases (*n* = 128,848) in human. Our results highlight that comparative epigenomics together with GWAS and selection signature provides novel biological insights into the phenotypic variation and adaptive selection in cattle. The large-scale genotype-phenotype associations in cattle could in turn contribute to biomedical researches in human, which may provide additional information beyond laboratory model organisms, particularly when more novel phenotypes become available in the near future.

## Results

### Cross-species mapping of epigenomes from human to cattle

**Fig. 2:**
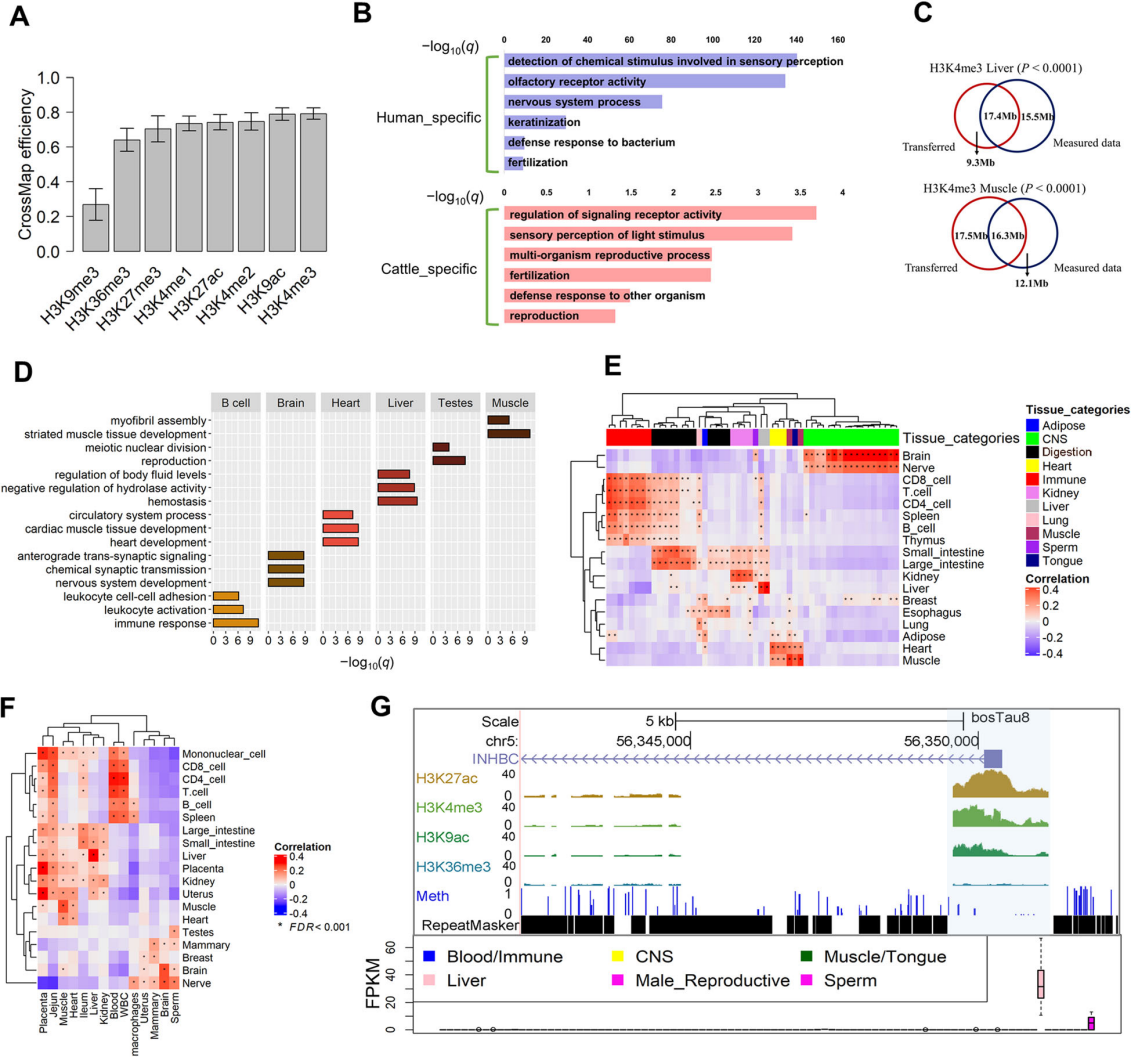
Cross-species mapping of epigenome data and validation of predicted epigenomes. **a** Transferring efficiency of eight histone marks from human to cattle using CrossMap. **b** Enriched Gene Ontology (GO) terms for genes that are not transferred from human (H3K4me3) to cattle (human-specific) and genes that are not covered by transferred epigenome (H3K4me3) in cattle (cattle-specific). **c** Venn plots for overlaps between length of transferred H3K4me3 peaks and that of measured ones in liver and muscle, respectively. **d** Enriched GO terms for genes with tissue-specific histone marks (H3K4me3) in six tissues. **e** Heat-map plot for correlations of *t*-statistics of genes based on expression and H3K4me3 signals (Methods). *X*-axis: tissues for gene expression. *Y*-axis: tissues for H3K4me3 signals. “*” denotes significant positive correlations after FDR correction (FDR < 0.001). **f** Heat-map plot for correlation of *t*-statistics of genes based on promoter DNA methylation and H3K4me3 signals. *X*-axis: tissues for promoter DNA methylation. *Y*-axis: tissues for H3K4me3 signals. “*” denotes significant positive correlations after FDR correction (FDR < 0.001). **g** An example of gene (*INHBC*) with liver-specific H3K4me3 signal. UCSC tracks included transferred H3K27ac, H3K4me3, H3K9ac, H3K36me3, and measured DNA methylation (Meth) data in cattle. Bottom is the gene expression (FPKM) of *INHBC* across 91 bovine tissues

To predict epigenomic states in cattle, we retrieved a total of 1300 human epigenomic datasets from public resources (i.e., ENCODE project [8] and the Epigenomic RoadMap [11]), covering 178 unique tissues and cell types. The analyzed data included ChIP-seq of eight histone modification marks: H3K4me3 (transcriptionally active promoters), H3K9ac (actively transcribed promoters), H3K27ac (active promoters or enhancers), H3K4me2 (active promoters or enhancers), H3K4me1 (active or primed enhancers), H3K36me3 (actively transcribed regions), H3K27me3 (polycomb repression), and H3K9me3 (heterochromatin). We observed that distributions of all predicted histone marks around transcription start sites (TSS) and transcription terminal sites (TTS) in cattle were similar to those originally observed in humans, providing evidences for the conservation of epigenomes between human and cattle (Additional file 1: Fig. S1A). Among these histone marks, H3K4me3 showed the highest transferring efficiency, of which an average of 79.2% was transferred from human to cattle, followed by H3K27ac (74.2%), while H3K9me3 had the lowest transferring efficiency, of which only 26.8% was transferred on average (Fig. 2a). These findings support that since the divergence of human and cattle from their common ancestor, their genome sequences of active regulatory elements (e.g., promoters and enhancers) have been evolutionarily conserved, whereas repeat-rich regions of constitutive heterochromatin have evolved rapidly. In the subsequent analyses, we thus focused on three evolutionarily conserved histone marks that have distinct functions on transcriptional activation, including H3K4me3, H3K27ac, and H3K36me3.

By clustering tissues and cell types with predicted histone marks in cattle, we identified similar patterns as those obtained from the original data in human studies (Additional file 1: Fig. S1B), suggesting that the transferred histone peaks were proper for subsequent analyses. The transferred histone peaks covered the majority of bovine genes (79.5%, 80.9%, and 86.9% for H3K4me3, H3K27ac, and H3K36me3, respectively) in at least one tissue or cell type. We found that genes, which were covered by transferred histone peaks in cattle, were more evolutionarily conserved (i.e., lower dN/dS ratio) than “species-specific genes,” defined as human-specific genes (*n* = 2163) that were not aligned to cattle, and cattle-specific genes (*n* = 3573) that were not covered by transferred peaks (Additional file 1: Fig. S1C). Based on gene functional analysis, human-specific genes were significantly (FDR < 0.05) engaged in olfactory reception, sensory perception, nervous system process, keratinization, and fertilization (Fig. 2b), which was consistent across all three histone marks (Additional file 1: Fig. S1D). In contrast, cattle-specific genes were significantly enriched in the regulation of signaling receptor activity, sensory perception of light stimulus, reproduction, and defense in response to other organisms (Fig. 2b, Additional file 1: Fig. S1D). These observations were in line with [29], which proposed that cattle-specific genes were involved in innate immunity, partially due to the substantial load of microorganisms present in the rumen of cattle. These genes also regulate ruminant-specific aspects of fetal growth, maternal adaptations to pregnancy, and the coordination of parturition [29]. In summary, these results may reflect the differences in adaptive evolution between human and cattle after their divergence of ~ 90 million years ago.

### Validation of predicted epigenome using experimentally generated epigenome, transcriptome, and methylome in cattle

#### ChiP-seq validation

By overlapping two predicted histone marks (H3K4me3 and H3K27ac) with their actual ChIP-seq data measured in the corresponding bovine tissues (i.e., liver and muscle) [30, 31], we validated 65.2%, 48.2%, and 64% of predicted H3K4me3 using measured data from one liver and two muscle samples, which covered 1.2%, 1.1%, and 1.2% of the cattle genome, respectively (Fig. 2c, Additional file 2: Fig. S2A), as more than expected (*P* value < 0.0001; 10,000 times permutation tests using R package: GAT [32]). In addition, we validated 34% of predicted H3K27ac using a measured dataset, which covered 3.7% of the cattle genome (*P* value < 0.0001) (Additional file 2: Fig. S2A). These findings reveal the ranges of sensitivity and specificity for our comparative epigenomic approaches between human and cattle.

#### RNA-seq validation

By grouping 1300 samples into 34 distinct categories based on their tissue similarity and known biology (Additional file 3: Table S1–3), we detected histone marks that were highly specifically enriched in each of these tissue categories. Our functional enrichment analysis showed that genes with tissue-specific histone marks were functionally concordant with the biology of target tissues (Fig. 2d). For instance, genes with B cell-specific H3K4me3 were significantly (FDR < 0.05) engaged in immune responses, while genes with brain-specific H3K4me3 were significant for nervous system development, heart for cardiac muscle tissue development, liver for the negative regulation of hydrolase activity, testes for reproduction, and muscle for striated muscle tissue development (Fig. 2d). More importantly, we observed that predicted tissue-specific epigenomes were significantly and positively correlated with tissue-specific gene expression in the corresponding tissues obtained from 723 RNA-seq data in cattle [33] (Fig. 2e). We used liver as an example in Additional file 2: Fig. S2B, which showed a significant correlation (Pearson’s correlation *r* = 0.57; *P* < 2.2e−16) between *t*-statistics (measurements of tissue specificity) of gene expression and those of predicted H3K4me3. Similar patterns were observed for H3K27ac and H3K36me3 (Additional file 2: Fig. S2C and D). These findings suggest that cross-species mapped histone marks are predictive for tissue-specific gene expression.

#### WGBS validation

We detected genes with tissue-specific hypomethylated promoters from 40 bovine WGBS datasets, including 20 tissues, and observed that they were significantly and positively correlated with predicted tissue-specific H3K4me3 and H3K27ac in the same or similar tissues (Fig. 2f, Additional file 2: Fig. S2F), supporting that hypomethylated promoters were associated with transcriptional activation. For instance, we observed that genes with liver-specific H3K4me3 showed the lowest DNA methylation levels in their promoters in liver as compared to all other 19 tissues (Additional file 2: Fig. S2E). We took *INHBC* gene as an example in Fig. 2g, which encodes a member of transforming growth factor-beta (TGF-β) protein superfamily and plays a role in hepatocyte proliferation [34]. *INHBC* highly and specifically expressed in cattle liver, and its promoter showed specifically high signal intensities of three predicted active histone marks (H3K4me3, H3K27ac, and H3K9ac) in liver as well. Correspondingly, we observed the low methylation level in its promoter region in liver (Fig. 2g). In contrast, we did not observe clear patterns between DNA methylation and H3K36me3 in gene bodies (Additional file 2: Fig. S2G), which was consistent with the fact that roles of DNA methylation in gene body remains elusive [35]. Collectively, these results suggest that cross-species mapped histone marks can predict tissue-specific DNA methylation, at least in promoters.

### Detection of trait- and selection-relevant tissues in cattle

**Fig. 3:**
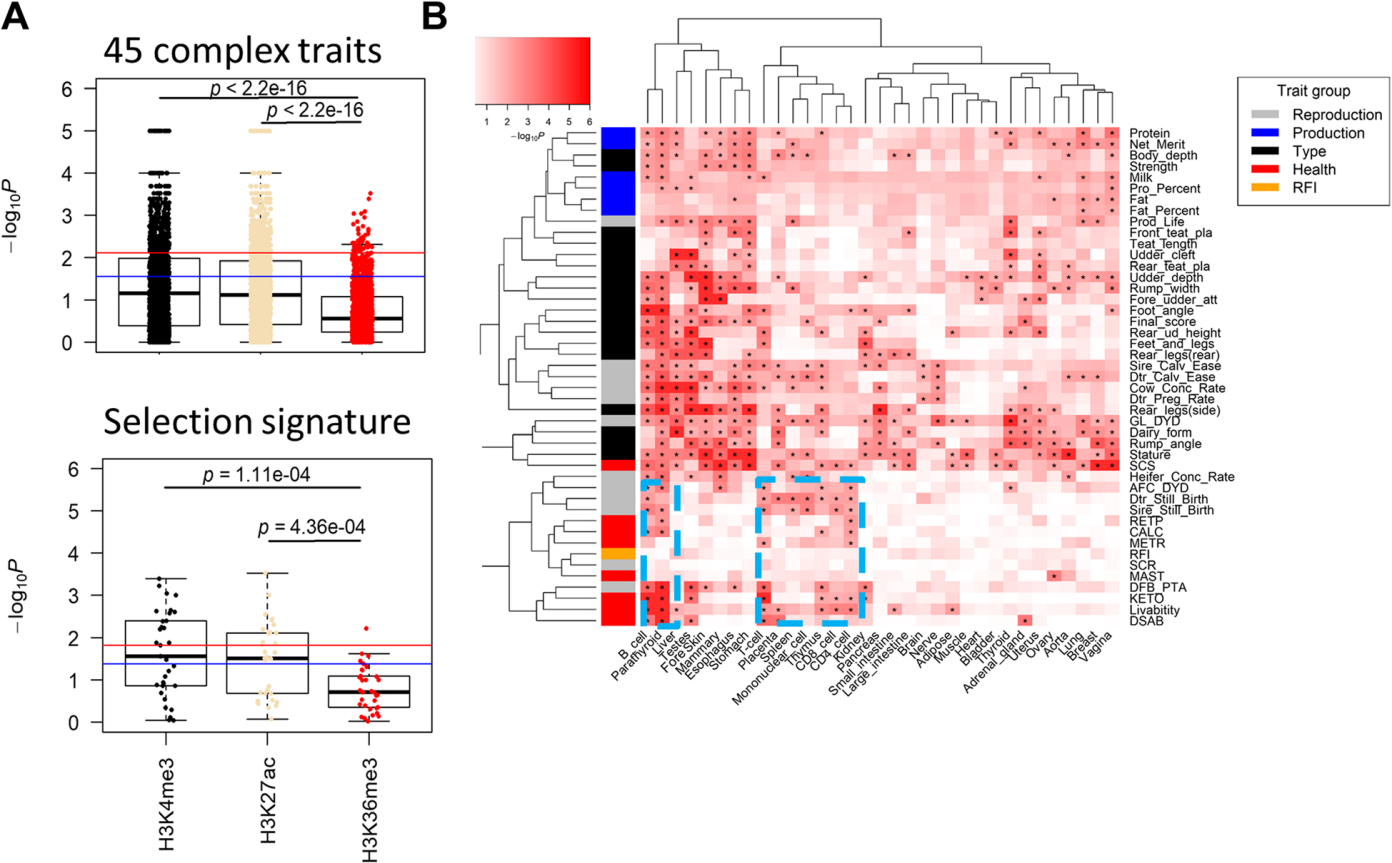
Enrichment analysis for 45 complex traits and selection signature in cattle. **a** Comparisons of GWAS (up) and selection signature (down) enrichments among tissue-specific H3K4me3, H3K27ac, and H3K36me3 (top 5%). Student *t* test was used to compare the enrichments between H3K4me3/H3K27ac and H3K36me3. Blue line denotes *P* < 0.05; red line denotes FDR < 0.05. **b** Association of tissues with complex traits based on GWAS signal enrichment analyses using tissue-specific H3K4me3 (top 5%). Blue boxes contain associations of health and reproduction traits with immune related tissues. “*” denotes FDR < 0.05

By conducting GWAS signal enrichment analyses with the detected tissue-specific histone marks for 45 complex traits of economic importance and selection signatures of beef vs. dairy cattle, we observed that tissue-specific H3K4me3 and H3K27ac showed a significantly higher enrichment (i.e., − log_10_*P*) of GWAS signals and selection signatures than tissue-specific H3K36me3 (Fig. 3a). This finding demonstrates that genomic variants associated with economic traits and adaptive evolution are more likely enriched in regulatory regions than protein-coding regions in cattle, which validates previous observations in human [36].

#### Trait-relevant tissues

Using a sum-based marker-set test (methods), we found that, among the 45 GWAS traits, most of them (*n* = 43) had at least one relevant-tissue (FDR < 0.05) (Fig. 3b). Production and body conformation traits were significantly associated with multiple tissues, reflecting their highly polygenic inheritance [37]. For instance, protein and milk yields were significantly associated with B cells, parathyroid, liver, stomach (rumen in cattle), lung, and vagina. Stature was significantly associated with aorta, muscle, foreskin, and multiple digestive tissues (e.g., esophagus, stomach, small intestine, large intestine), which was in line with human height that was proposed to associate with multiple tissues, including muscle, cardiovascular, and digestive tissues [12, 14, 38]. Of interest, all health traits and most of reproduction traits had significant enrichments in placenta and immune/blood-related cell types/tissues, such as B cell, T cell, spleen, and thymus (Fig. 3b). This is in agreement with previous findings in human that reproduction traits have been linked with immunological processes such as allergic response to sperm and immunological tolerance of the embryo [39]. We observed that mammary is the top significant tissue for somatic cell score (SCS, an indicator of mastitis), consistent with prior knowledge that an infection of the udder is the main cause for increased somatic cell counts in milk [40]. Vagina is another tissue significantly associated with SCS, which might suggest a shared molecular mechanism underlying host responses to bacterial infection between vagina and mammary. Of special interest were brain and nerve, which were the relevant tissues for five fertility traits, including sire calving ease, daughter calving ease, cow conception rate, daughter pregnancy rate, and gestation length. This might reflect that the brain plays a role in the regulation of hormonal complexity during pregnancy [41]. For instance, the pituitary gland, a brain region, is one of the most affected organs and enlarged due to lactotroph hyperplasia [42].

#### Selection-relevant tissues

**Fig. 4:**
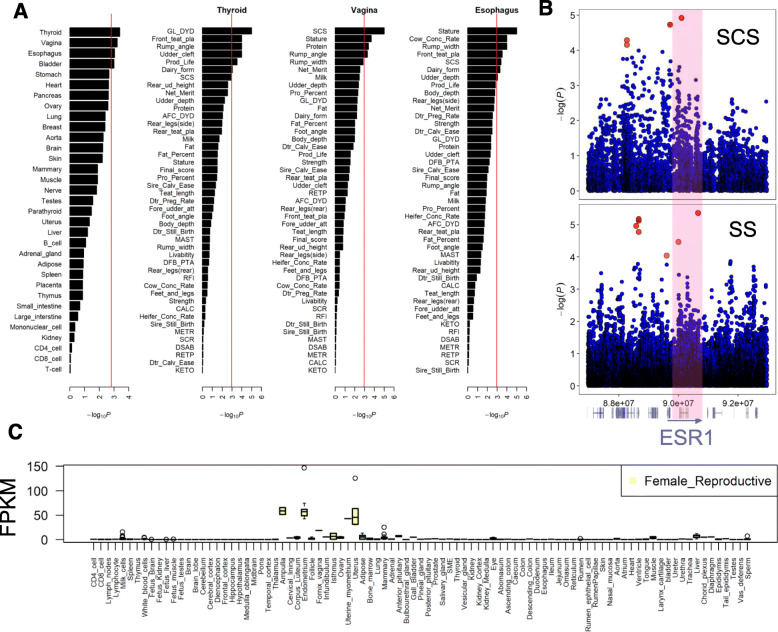
Enrichment of selection signature across multiple tissues. **a** The first bar plot is for enrichment of selection signature in tissue-specific H3K4me3 across multiple tissues. The second, third, and fourth ones are for associations of traits with thyroid, vagina, and esophagus, respectively, based on GWAS signal enrichments of tissue-specific H3K4me3. The red line denotes FDR < 0.05. **b** An example of shared peaks between selection signature (SS) and GWAS of somatic cell scores (SCS). *ESR1* gene, which shows tissue-specific H3K4me3 signals in vagina, locates within the peak. **c** Gene expression of *ESR1* in 91 bovine tissues

We retrieved selection signatures from [43]. They were detected between 8 dairy and 7 beef cattle breeds with a linear mixed-model approach using Run 6 of the 1000 Bull Genomes Project. We observed significant enrichments of selection signatures of beef vs. dairy cattle in tissue-specific H3K4me3 of thyroid, vagina, esophagus, and bladder (Fig. 4a). Gestation length was the top significant trait for thyroid, which agreed with that Holstein-Friesian cows have shorter gestation length than continental beef breeds like Charolais, Limousin, and Simmental and British beef breeds like Angus, Hereford, and Shorthorns [44]. In addition, this finding indicates the importance of thyroid hormone on pregnancy and embryonic development, as the maternal thyroxine (T4) and triiodothyronine (T3) play key roles in normal growth and development of the fetus [45, 46]. For vagina, SCS was the top significant trait, consistent with that intensive selection for milk production in dairy cattle has dramatically reduced their immune ability as compared to beef cattle [47]. For esophagus, stature was the top significant trait, in line with the inverse association between height and the risk for esophagus diseases [48]. This may partially explain the deviation of body size between beef and dairy cattle [49]. We identified a gene of interest, estrogen receptor 1 (*ESR1*), which showed tissue-specific H3K4me3 peaks in promoter for vagina. *ESR1* was associated with both SCS and selection of beef vs. dairy cattle (Fig. 4b), which were highly expressed in female reproductive tissues (vagina, ovary, uterus) and moderately expressed in milk cell and mammary gland (Fig. 4c). We observed concordant regions near the *ESR1* gene for both SCS GWAS signals and selection signature (SS) signals. Significant signals outside the gene may be in LD with the signals inside the gene. Estrogen receptor plays a role in regulation of inflammatory response [50] and is associated with udder infection [51]. All these findings imply that *ESR1* might be under divergent selection between beef and dairy cattle and it may drive the SCS divergence, due to its regulation of the estradiol level in response to inflammatory infection.

### Detection of trait-relevant tissues in human

**Fig. 5:**
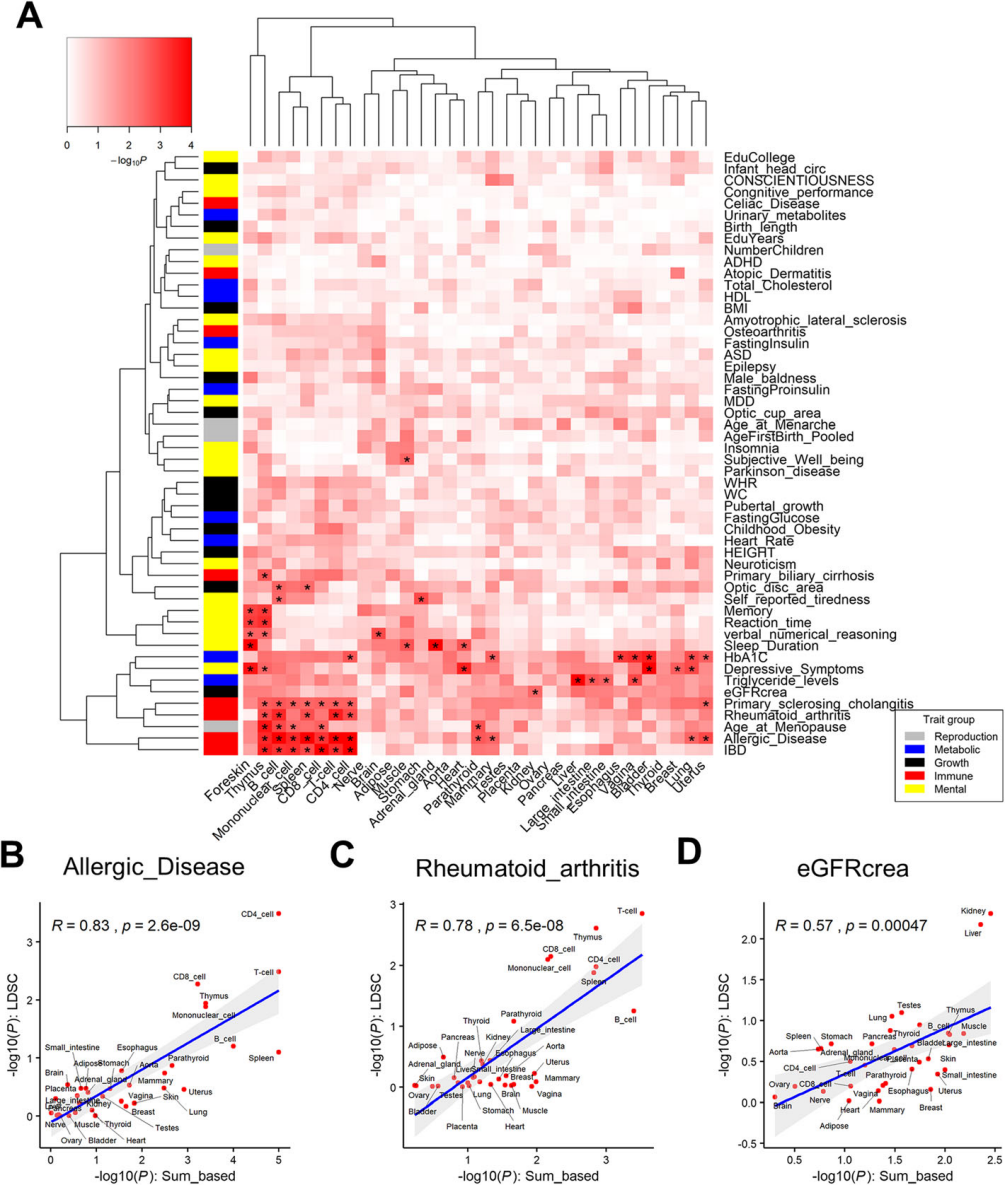
Associations of 33 tissues with 58 complex traits and diseases in human. **a** The enrichments (− log_10_*P*) were obtained from GWAS enrichment analysis with tissue-specific H3K4me3 (top 5%) using the sum-based marker-set test. “*” denotes FDR < 0.05. **b**–**d** Pearson correlations of enrichments (− log_10_*P*) between the stratified LD score regression (LDSC) and sum-based marker-set test

We utilized the same sum-based marker-set test to detect relevant tissues for 58 complex traits and diseases in human using tissue-specific histone marks (Fig. 5a). To validate our method, we applied the commonly used stratified LD score regression (LDSC) to detect trait-relevant tissues in human as well [52]. Results from sum-based marker-set test and LDSC were significantly and positively correlated. For example, immune-related diseases, like allergic disease and rheumatoid arthritis, were highly and significantly correlated (*r* = 0.83 *P* = 2.6e−09 and *r* = 0.78; *P* = 6.5e−08), between the two methods (Fig. 5b, c). Another example is the estimated glomerular filtration rate based on serum creatinine (eGFRcrea), of which the top significant tissue is kidney from both methods (*r* = 0.57; *P* = 4.7e−04) (Fig. 5d). Therefore, our results from sum-based marker-set method were comparable to those from previous studies, shedding light on trait-relevant tissues. For instance, kidney was the top significant tissue for eGFRcrea, which is the best measurement for kidney function [53]. Liver was the most significant tissue for triglyceride levels, consistent with that liver plays key roles in producing triglycerides. Adrenal gland was the most significant tissue for sleep duration, which corresponds to the essential role of cortisol secreted by the adrenal gland in controlling circadian rhythm [54]. For verbal numerical reasoning, brain was the most relevant tissue, which is consistent with our understanding for brain control of intelligence. Consistent with previous studies [13, 38, 52], we observed that blood/immune tissues were significantly relevant with multiple immune diseases and a reproduction trait, age at menopause. The involvement of age-at-menopause in blood/immune tissues might suggest that sex steroids play roles in immune responses [55], and the immune system triggers a woman’s biological clock as well [56]. Additionally, we observed that muscle was significantly associated with subjective well-being, which might suggest the role of physical exercise in the promotion of health and human well-being [57].

### Comparison of complex traits and diseases between cattle and human

**Fig. 6:**
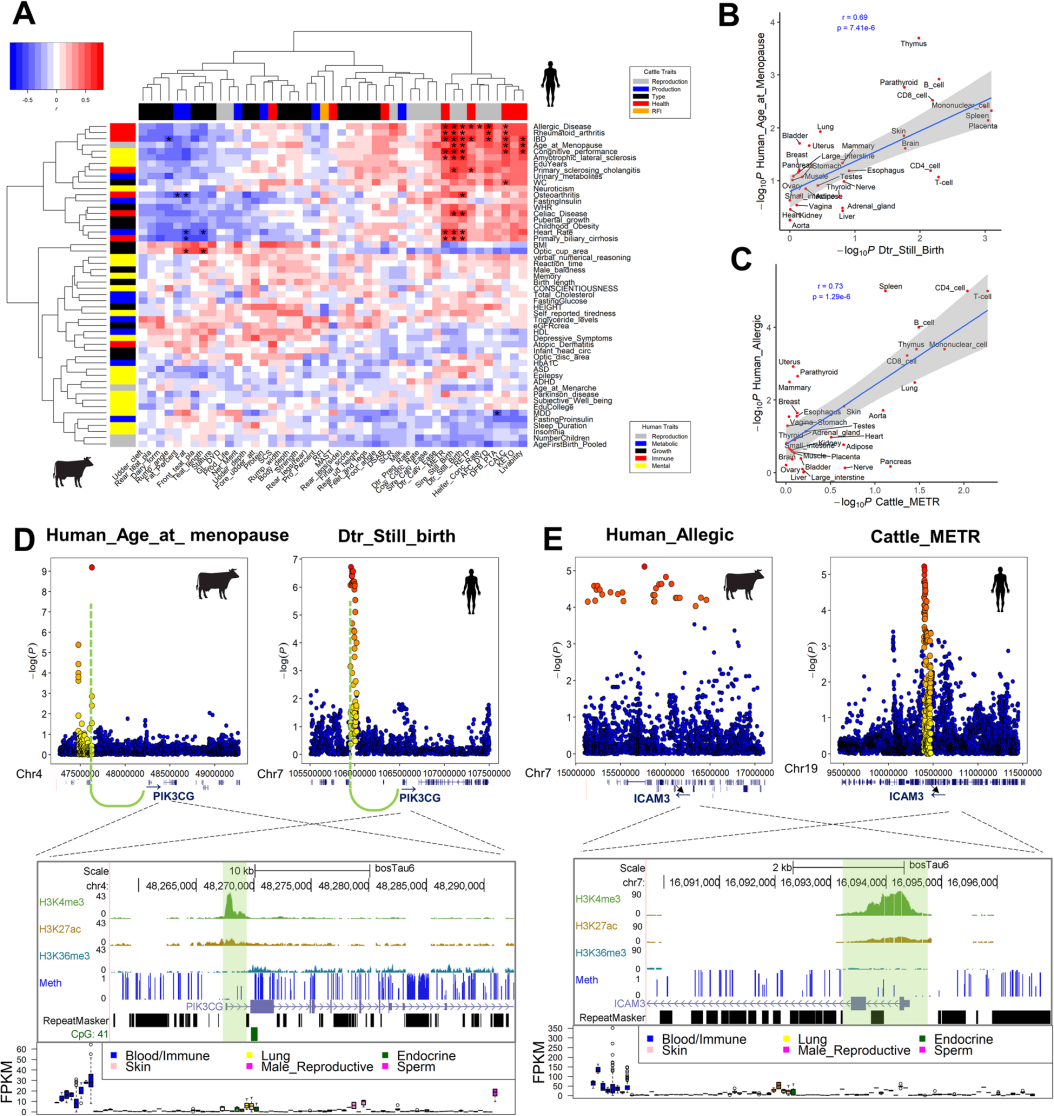
Shared genetic architecture between human diseases/traits and cattle traits. **a** Correlations of complex traits between human and cattle based on GWAS signal enrichment of tissue-specific H3K4me3 (top 5%) across all tested tissues. “*” denotes FDR < 0.05. **b** Correlation between age at menopause in human (Human_Age_at_menopause) and daughter still birth (Dtr_Still_Birth) in cattle. **c** Correlation between allergic disease (Human_Allergic) in human and metritis (Cattle_METR) in cattle. **d**, **e** Examples of two genes, *PIK3CG* and *ICAM3*, with tissue-specific H3K4me3 in mononuclear cells and T cells, respectively. Dots plots (up) are GWAS peaks within 1 Mb regions of the genes in cattle and human. UCSC browser tracks of genes (down) include transferred H3K27ac, H3K4me3, H3K9ac, H3K36me3, and measured DNA methylation (Meth) data in cattle. Below are the tracks for gene expressions of *PIK3CG* and *ICAM3* in 91 bovine tissues. Green curve lines in **d** indicate a capture Hi-C contact between *PIK3CG* and GWAS signal peaks (~ 610 kb upstream) in human and cattle

To genetically compare complex traits/diseases between human and cattle, we estimated 2610 trait correlations by using their tissue-trait associations. We observed that a fertility trait (age-at-menopause) and several immune diseases in human, such as inflammatory bowel disease (IBD), allergic disease, and rheumatoid arthritis, were significantly (FDR < 0.05) correlated with multiple reproduction and health traits in cattle (Fig. 6a). For instance, the age-at-menopause in human was significantly (*r* = 0.69; *P* = 7.41e−06) correlated with daughter-still-birth in cattle (Fig. 6b), and blood/immune tissues were the most relevant tissues for both traits. We found that *PIK3CG* gene, which showed specific expression and histone marks in blood/immune tissues, had significant GWAS peaks (within 1 Mb upstream) for both age-at-menopause in human and daughter-still-birth in cattle (Fig. 6d). *PIK3CG* mainly functions in immune, inflammatory, and allergic responses [58, 59]. In addition, a previous study proposed that non-synonymous variants of *PIK3CG* might regulate the fragile X-associated primary ovarian insufficiency associated with woman’s reproductive life span [60]. Noticeably, the lead SNP was located in a capture Hi-C contact (chr7: 105993268-106000857) with promoter of *PIK3CG* in human. We further validated that the lead SNP was located in the enhancer region across multiple immune-relevant cell types, such as primary monocytes from peripheral blood, primary neutrophils from peripheral blood, and primary B cells from cord blood [11]. All these indicated that the causal variant within this QTL region physically interacts with *PIK3CG* in a long-range enhancer-promoter means, which further influences age-at-menopause by regulating the activity of this particular gene. We found that the Hi-C contact was conserved between human and cattle, which also linked the lead SNP of daughter-still-birth with *PIK3CG* in cattle (Fig. 6d). Furthermore, amino acid sequence alignment in multi-species showed the high conservation of PIK3CG protein (Additional file 4: Fig. S3), indicating similar functions of this gene among all mammals.

Another example of interest was two immune-relevant traits, human allergic disease and cattle metritis, which were significantly (*r* = 0.73; *P* = 1.29e−06) correlated with each other (Fig. 6c). *ICAM3* gene, which showed specific expression and histone marks in blood/immune tissues, had significant GWAS peaks for both allergic disease in human and metritis in cattle (Fig. 6e), consistent with the indispensable role of *ICAM3* in immune response and its association with lymphocyte, monocyte, neutrophil percentage, and rheumatoid arthritis in the UK Biobank [61] (region PheWAS, set region ± 50 kb; *P* value threshold 1e−8).

Furthermore, we noted that several reproduction traits in cattle, such as sire-still-birth and heifer-conception-rate, could be useful for studies in human fertility, because such traits were hard to measure in human population. More interestingly, we observed several human immune diseases significantly (FDR < 0.05) correlated with such reproduction traits in cattle. The role of immune system in the reproduction processes has been discussed widely in human [62, 63]. For instance, the human female reproductive tract acts as an initial barrier to foreign antigens, of which two common samples are fetal allograft and semen/sperm [64]. Taken all together, these suggested that the genetic architecture of complex traits in cattle, particularly for health and reproduction traits, could be a good model for understanding similar traits in human.

## Discussion

In this study, we demonstrated and validated that cross-species extrapolation of epigenome could capture tissue-specific patterns of gene expression and DNA methylation in cattle. By integrating predicted tissue-specific histone marks with large-scale GWAS and selection signature of beef vs. dairy, we identified relevant tissues for 45 economically important traits and explored the artificial selection in cattle. By investigating the genetic similarities of complex traits between cattle and human, we proposed that cattle could be a potential model organism to guide human biomedical researches beyond laboratory model organisms (e.g., mice and fruit fly), especially for immune and reproduction traits. To our best knowledge, this is the first publication to integrate comparative epigenomics with large-scale genotype-phenotype associations to gain insights into the underlying molecular mechanisms of complex traits and artificial selection in animals and to explore their genetic similarities with human complex traits and diseases. Collectively, our results demonstrate the potential of comparative epigenomics to explore the biological and genetic basis of phenotypic diversity and adaptive evolution in non-model animals through borrowing regulatory annotations from well-established organisms.

Cross-species comparison has been widely used to explore epigenetic evolution. By comparing 13 distinct histone marks and transcriptional regulators in pluripotent stem cells among human, mouse and swine, Xiao et al. showed that these epigenetic signals were largely conserved across species [19], indicating that comparative epigenomics could be a tool for annotation of regulatory elements in other species that are lack of such information. Zhou et al. reported that the conservation of tissue-specific DNA methylation among human, mouse, and rat were likely driven by their primary sequence conservation [22]. Ebert et al. reported that the cross-species mapping of epigenomes (i.e., H3K4me3, H3K27ac, and H3K36me3) in six tissues/cell types from human and/or mouse to other 13 species enabled them to predict gene expression in the target species [23]. Here, through analyzing 723 transcriptomes and 40 methylomes, we comprehensively demonstrated that the cross-species mapping of histone marks were not only predictive for tissue-specific gene expression but also for tissue-specific DNA methylation. Together, these findings suggested the potential of utilizing the available epigenome data from reference species to initiatively annotating a range of target species.

In human, genomic variants associated with complex traits have been widely reported to be concentrated in regulatory elements rather than protein-coding regions [36, 65]. In this study, we confirmed this in cattle by observing a higher enrichment of GWAS hits and selection signals in tissue-specific promoters (i.e., H3K4me3 and H3k27ac) than in tissue-specific gene body regions (i.e., H3K36me3). This was in agreement with our recent study that found GWAS signals and selection signature were significantly enriched in chromatin states relevant with active promoters and enhancers in cattle [16]. A previous study on sheep also reported that *cis*-regulatory elements contributed to the adaptive evolution of modern breeds by mapping selection signatures to chromatin states that were cross-species mapped from human [66]. Here, through integrating predicted tissue-specific histone marks with GWAS and selection signature, we systematically identified tissues and cell types that were relevant with 45 complex traits and artificial selection in cattle, providing novel insights into their biological underpinnings. We thereby propose that cross-species mapping of epigenomes from reference species (human and mice) to a large number of target species could be a powerful way to biologically explaining genotype-phenotype associations and adaptive evolution in the target species, which is still lack of epigenomes across multiple tissues and cell types.

Over the past 100 years, diverse phenotypes and comprehensive pedigrees have been routinely and accurately recorded in the cattle industry [24, 67]. Strong artificial selection on economically important traits has been observed in cattle, such as selection for growth rate and milk production in beef and dairy cattle, respectively [68, 69]. Due to the negatively genetic correlation between fitness traits (e.g., health and fertility) and production traits (e.g., milk), the strong artificial selection on production traits increase not only the beneficial alleles for production but also the deleterious alleles for fitness traits. Compared to nature populations, such as human, where deleterious alleles of large effects are rare due to strong natural purifying [70], this strong artificial selection in cattle could be useful for dissecting genetic variants underlying fitness traits, subsequently providing valuable information for biomedical researches in human [68, 69]. Furthermore, the strong artificial selection led to a relatively small effective population size of cattle, which much easily exposed recessive deleterious mutations [25]. In addition, by thoroughly breaking down the linkage disequilibrium (LD) blocks, cross-species comparison is more effective in targeting causal genes/variants than within species, given that similar complex outcomes shared causal variants among species.

We noted several limitations in the current study. First, we focus on histone marks, while topologically associating domains (captured by Hi-C), chromatin accessibility patterns (captured by ATAC-seq), and others were widely conserved across species [71, 72]. It could be of interest to explore them together with histone marks in the near future, as the increasing availability of such data sets across multiple tissues and cell types in human. Second, the sequence conservation may not be equivalent to functional conservation. In other words, the signal intensities of epigenomes in the aligned sequence might be different between two species being studied. In addition, the cross-species mapping of epigenomes only enabled us to explore the conserved regions, ignoring species-specific regions and thus limiting sensitivities and specificities of our approaches. With more epigenomes becoming available from FAANG project in the future [15], it could be possible to study other regulatory elements, like enhancers which were often species-specific [31]. Third, the trait-relevant tissues detected in this study are “proximal” tissues not exactly causal tissues, similarly as significant GWAS hits, which are often not the causal variants for the studied traits. Further experimentally functional validations are required to confirm the causal tissues and cell types for a particular casual variant of a complex outcome.

## Conclusions

Collectively, our study “borrowed” histone marks in multiple tissues from human to functionally annotate the cattle genome. In combination of selection signatures and GWAS datasets, our results reflect the adaptive evolution and trait-relevant tissues in cattle. Comparative epigenomics between cattle and human sheds light on the potential that cattle serves as a model for human complex traits.

## Methods

### Cross-species mapping of large-scale epigenome data from human to cattle

In total, we downloaded narrow-peak files (bed format) of 8 histone marks from 1300 samples from ENCODE and Roadmap projects (https://www.encodeproject.org/), covering 178 unique tissues and cell types. We occasionally grouped tissues from different parts of the same organ into one category. For example, we consider heart left ventricle, heart right ventricle, and cardiac atrium as one tissue, heart. We excluded ESC, IPSC, and ES-derived cell lines to focus on the somatic tissue/cell types. Finally, we obtained 33 tissue categories and an “other” category (details in Additional file 3: Table S1–3). All the human epigenomic data are based on GRCh38/hg38. We thus downloaded the whole genome alignments between human (GRCh38/hg38) and cattle (UMD3.1.1) from the UCSC Genome Browser in the form of the chain files and processed as described in the UCSC Genome Wiki website (http://genomewiki.ucsc.edu/index.php/HowTo:_Syntenic_Net_or_Reciprocal_Best) to derive reciprocal best chains. We further used crossMap 0.3.0 [73] to build pairwise symmetric alignment blocks with default parameters.

### Detecting tissue-specific histone marks

We obtained the annotation files for human (GRCh38/hg38 and GRCh37/hg19) and cattle (UMD3.1.1) from Ensembl database (https://www.ensembl.org). We only kept protein-coding genes for subsequent analyses. We defined promoter regions as 1.5 kb windows around the transcriptional start site (from − 1000 bp to + 500 bp). For gene body regions, we excluded genes with length less than 750 bp. For H3K4me3 and H3K27ac, we obtained their histone signal intensities in gene promoters. For H3K36me3, we calculated histone signal intensities in gene bodies by adjusting the length of transcribed region for each gene. We assigned 33 tissue categories into 23 categories based on the tissue similarity (Additional file 3: Table S1–3). We excluded samples from the same tissue class [52], when calculating the *t*-statistics of a histone mark for a tissue category which measured the tissue specificity for the particular histone mark. For instance, when detecting B cell-specific histone marks, we excluded all samples belonging to the immune-blood system, such as T cell and thymus. We fitted the following linear regression model to detect tissue-specific histone marks.

$$ \mathbf{y}=\boldsymbol{\upmu} +\mathbf{X}b+\mathbf{e} $$

where y is the vector of histone signal intensities in gene promoter (H3K4me3 and H3K27ac) or gene body (H3K36me3); μ is the intercept; X is the dummy variable for tissue, where we assigned “1” for samples from the tissue categories being tested and “− 1” for the remaining samples; *b* is the tissue effect; and **e** is the residual effect. We fitted the model using the ordinary least-square approach implemented in R and calculated *t*-statistics as *b* divided by its standard error. In each tissue category, we considered top 3%, 5%, and 10% of histone marks as tissue-specific, respectively, based on the ranking of *t*-statistics.

### Detecting tissue-specific genes using RNA-seq data in cattle

We uniformly analyzed 723 RNA-seq datasets in cattle as described in (http://cattlegeneatlas.roslin.ed.ac.uk/). To detect tissue/cell type specific genes, we computed a *t*-statistics of each gene in each of 91 tissue/cell types after correcting for known batch effects. We scaled the log_2_-transformed expression (i.e., log_2_FPKM) of genes to have a mean of zero and variance of one within each tissue and cell type.

$$ \mathbf{y}=\upmu +\mathbf{X}b+\mathbf{Z}\mathrm{c}+\mathbf{e} $$

where **y** is the vector of scaled log_2_FPKM; μ is the intercept; **X** is the dummy variable for tissue, where samples of the tested tissue (e.g., CD4 cells) were denoted as “1,” while samples outside the same category (e.g., non-blood/immune tissues and cell types) as “− 1”; *b* is the tissue effect; Z is the matrix for co-variables, including age, sex, and study effects; c is the effects for co-variables; and **e** is the residual effect. We fitted this model for each gene in each tissue using the ordinary least-square approach, as implemented in R [74], and then obtained the *t*-statistics for each gene to measure its expression specificity in the corresponding tissue. To obtain relationships between predicted tissue-specific histone marks and measured tissue-specific expression, we correlated their *t*-statistics across shared genes (*n* = 19,746).

### Bioinformatics analysis of DNA methylation data

In total, we uniformly analyzed 40 WGBS datasets, including 20 distinct tissues (Additional file 5: Table S4). Briefly, we used FastQC v 0.11.2 and Trim Galore v 0.4.0 (--max_n 15) to check the read quality and filter the sequences, respectively. We subsequently mapped clean reads to the reference genome (UMD3.1.1) using Bismark software (0.14.5) with default parameters. We extracted the methylcytosines using *bismark_methylation_extractor* (--ignore_r2 6) function after de-duplicating duplicated reads. For each sample, we calculated DNA methylation level in gene promoters using a weighted methylation method as described in [75]. We computed *t*-statistics for tissue-specificity of promoter methylation as described above. Because DNA methylation in promoters was negatively correlated with gene expression, we thus assigned “− 1” for samples from a given tissue and “1” for the remaining samples to detect genes with tissue-specific low methylation in promoters.

### GWAS enrichment analysis based on tissue-specific histone marks

We collected GWAS summary statistics of 45 complex traits in cattle [24, 76]. We generally grouped these 45 complex traits into 5 categories, including reproduction (*n* = 12), production (milk-relevant; *n* = 6), body type (*n* = 17), health (immune-relevant; *n* = 9), and feed efficiency (residual feed intake, RFI; *n* = 1) traits. Details of the single-marker GWAS for body type, reproduction, and production traits using 27,214 US Holstein bulls were presented previously [24]. GWAS for health traits using ~ 10,000 bulls could be found in [76], while GWAS for feed efficiency using 3947 Holstein cows (i.e., RFI) were described by Li et al. [77]. For human GWAS data, we obtained the summary statistics for 58 complex traits with an average sample size of 128,848 and an average SNP number of 5,905,874. Details of human GWAS studies are summarized in [26]. We grouped these diseases and traits into 5 categories similarly as in cattle: reproduction (*n* = 4), metabolic (*n* = 13), mental (*n* = 22), immune (*n* = 8), and growth (*n* = 13). When integrating tissue-specific histone marks with human GWAS, we first transferred tissue-specific histone marks from GRCh38 to GRCh37 using UCSC LiftOver tool (-minMatch = 0.8), as coordinates of variants in GWAS were based on GRCh37. Detailed procedures were presented in [26]. We applied a sum-based marker-set test approach, implemented in the QGG package [78], to determine whether GWAS signals were enriched in tissue-specific histone marks. This approach employed a 10,000-time circular genotype permutation procedure and showed a better or at least equal performance compared to most of commonly used marker-set test methods in livestock [79–82], fruit fly [83], and human [84]. We found that enrichment results of GWAS signals and selection signatures were highly correlated (Pearson’s *r* ranging from 0.64 to 0.83) among three different cutoffs of tissue-specific histone marks, i.e., top 3%, 5%, and 10% (Additional file 6: Fig. S4A and B). In addition, results of GWAS enrichment were significantly correlated among all three histone marks across all tested traits, and the highest correlation (Pearson correlation *r* = 0.48 and *P* < 2e−16 for GWAS) were observed between H3K4me3 and H3K27ac (Additional file 6: Fig. S4C). We observed similar results for selection signature (Additional file 6: Fig. S4D). We thus showed results for top 5% of H3K4me3 in the “Results” section.

### Other downstream bioinformatics analyses

We used Genomic Association Tester (GAT) [32] to do the permutation tests (10,000 times) for computing the significance of overlaps between predicted epigenomes and measured epigenomes. We conducted gene functional annotation analyses for a list of genes using R packages, clusterProfiler [85], and GO terms with threshold of FDR < 0.05 were considered as significant ones. To assess whether GWAS peaks overlapped with chromosomal contact as measured using Hi-C data, we used promoter-capture Hi-C data with interaction score of contacts greater than 5 [86].

## Supplementary information

**Additional file 1: Figure S1.** Summary of epigenome data and predicted epigenomes. (A) Distributions of actual and predicted histone peaks around transcription start sites (TSS) and transcription terminal sites (TTS) in human and cattle. (B) Hierarchical clustering of tissues based on H3K27ac signals in gene promoters. (C) The ratio of non-synonymous to synonymous substitutions (dN/dS) of mapped histone mark peaks (Common), un-transferred regions in human (human-specific), and uncovered sequences by transferred epigenome in cattle (Cattle-specific). (D) Enriched GO terms for human-specific genes and cattle-specific genes.

**Additional file 2: Figure S2.** Cross-species extrapolation of epigenome data and validation of predicted epigenomes. (A) Venn plots for length of transferred and measured H3K27ac (H3K4me3) peaks in liver (muscle). (B) Pearson correlation (*r* = 0.57) between *t*-statistics of genes for gene expression and H3K4me3 signals in liver. (C) and (D) Correlations of *t*-statistics of genes based on RNA-seq and H3K27ac or H3K36me3 signals. X axis: tissues for gene expression. Y axis: tissues for H3K27ac or H3K36me3 signals. “*” denotes significant positive correlations after FDR correction (FDR < 0.001). (E) DNA methylation of the top 5% genes with liver-specific H3K4me3 in 20 tissues. (F) and (G) Correlations of *t*-statistics of genes based on promoter DNA methylation and H3K27ac or H3K36me3 signals. X axis: tissues for promoter DNA methylation. Y axis: tissues for H3K27ac and H3K36me3 signals. “*” denotes significant positive correlations after FDR correction (FDR < 0.001).

**Additional file 3: Table S1–3.** The summary list of epigenome data downloaded from ENCODE and RoadMap projects for H3K4me3 (Table S1), H3K27ac (Table S2) and H3K36me3 (Table S3).

**Additional file 4: Figure S3.** Alignment of the protein sequences of *PIK3CG* across different species.

**Additional file 5: Table S4.** The summary list of WGBS samples.

**Additional file 6: Figure S4.** Correlations of enrichments among three criteria and three histone marks. Correlations of enrichments for GWAS signals (A) and selection signature (B) among top 3%, top 5%, and top 10% genes with tissue-specific H3K4me3, respectively. Correlations of enrichments for GWAS signals (C) and selection signature (D) among three histone marks (top 5%).

## Data Availability

The cattle gene atlas (including FPKM of all 24,616 Ensembl genes across 723 samples) is publicly available at http://cattlegeneatlas.roslin.ed.ac.uk/. Peaks of ChIP-seq data were downloaded from GSE61936 for muscle H3K4me3 [30] and ERP006568 for liver H3K4me3 and H3K27ac [31]. The promoter capture Hi-C data was downloaded from CHiCP [87] (https://www.chicp.org/). The GWAS summary statistics for cattle complex traits are publically available through Figshare (https://figshare.com/s/ea726fa95a5bac158ac1; https://figshare.com/s/94540148512dddf7ed32). Selection signature is available in [43]. Details of human GWAS summary statistics are summarized in [26]. Among 40 WGBS data being analyzed, 4 WGBS of macrophages were downloaded from GSE110412 [88]; 2 breast WGBS samples from SRP190079; 6 WGBS of brain, mammary, and whole blood cells (WBC) from GSE106538 [89]; and 2 WGBS of sperm from GSE131851. A total of 26 WGBS data were newly generated with accession numbers GSE147087 (*n* = 24) and GSE147184 (*n* = 2; bone).

## Abbreviations

WGBS: Whole genome bisulfite sequencing
GWAS: Genome-wide association studies
FAANG: Functional Annotation of Animal Genomes consortium
TSS: Transcription start sites
TTS: Transcription terminal sites
LDSC: Stratified LD score regression
SCS: Somatic cell score
eGFRcrea: The estimated glomerular filtration rate based on serum creatinine
