# Supplementary figures and images for: Epigenomics and genotype-phenotype association analyses reveal conserved genetic architecture of complex traits in cattle and human
Source: BMC Biol. 2020 Jul 3;18:80. doi: 10.1186/s12915-020-00792-6 (PMC7334855; doi:10.1186/s12915-020-00792-6)

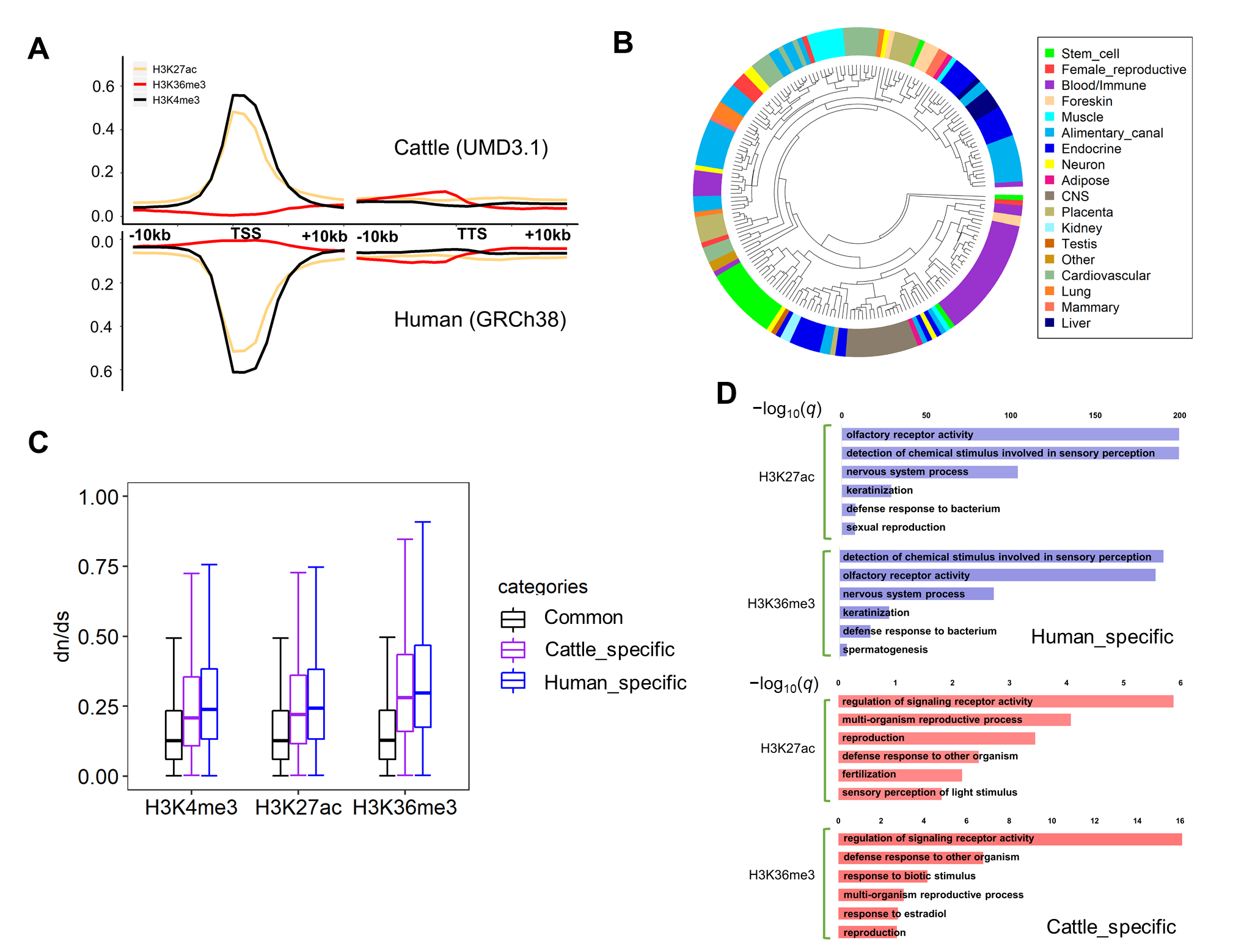

Supplement: Supplementary file 1 — Additional file 1: Figure S1. Summary of epigenome data and predicted epigenomes. (A) Distributions of actual and predicted histone peaks around transcription start sites (TSS) and transcription terminal sites (TTS) in human and cattle. (B) Hierarchical clustering of tissues based on H3K27ac signals in gene promoters. (C) The ratio of non-synonymous to synonymous substitutions (dN/dS) of mapped histone mark peaks (Common), un-transferred regions in human (human-specific), and uncovered sequences by transferred epigenome in cattle (Cattle-specific). (D) Enriched GO terms for human-specific genes and cattle-specific genes. [file 12915_2020_792_MOESM1_ESM.tif]

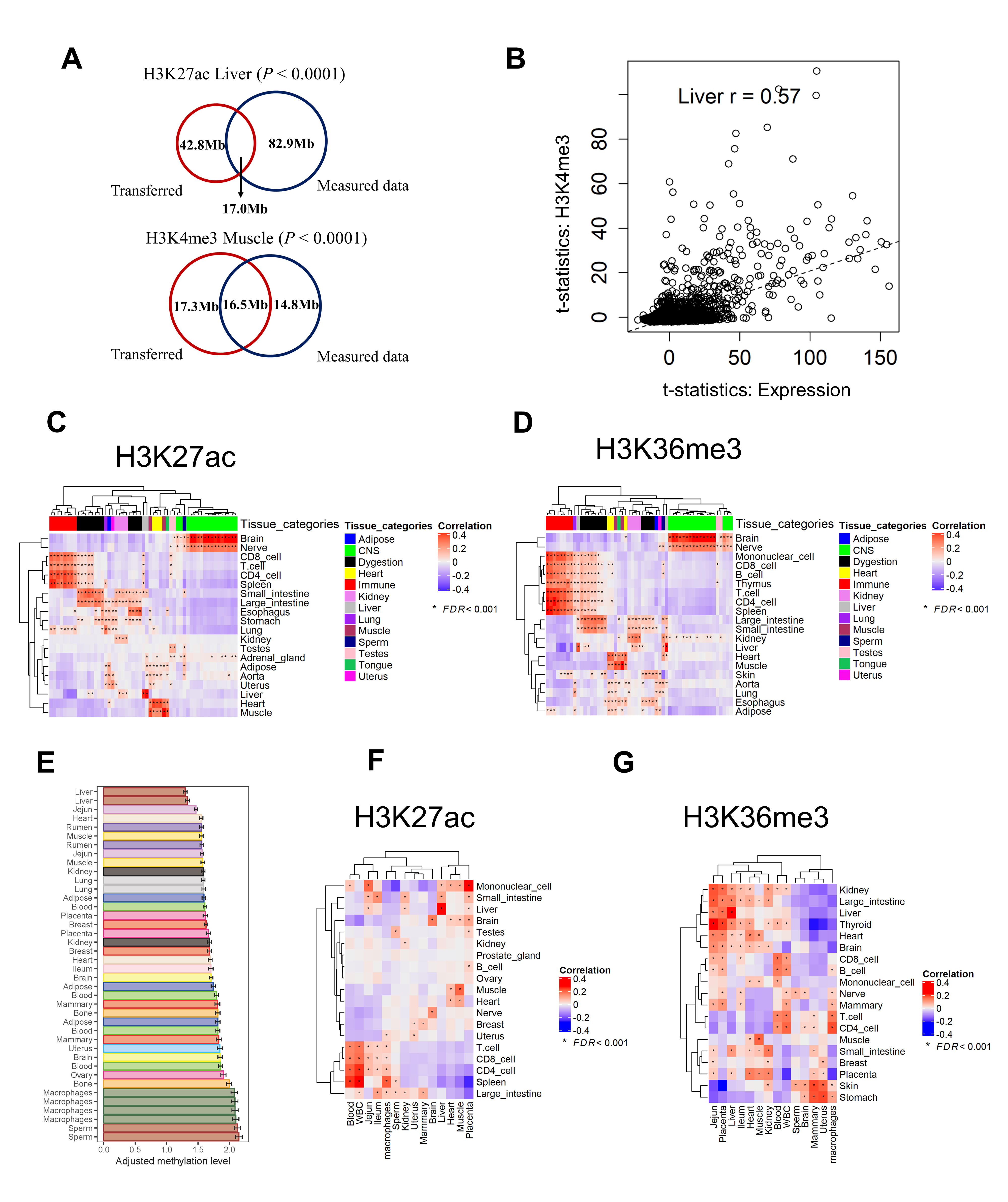

Supplement: Supplementary file 2 — Additional file 2: Figure S2. Cross-species extrapolation of epigenome data and validation of predicted epigenomes. (A) Venn plots for length of transferred and measured H3K27ac (H3K4me3) peaks in liver (muscle). (B) Pearson correlation (r = 0.57) between t-statistics of genes for gene expression and H3K4me3 signals in liver. (C) and (D) Correlations of t-statistics of genes based on RNA-seq and H3K27ac or H3K36me3 signals. X axis: tissues for gene expression. Y axis: tissues for H3K27ac or H3K36me3 signals. “*” denotes significant positive correlations after FDR correction (FDR < 0.001). (E) DNA methylation of the top 5% genes with liver-specific H3K4me3 in 20 tissues. (F) and (G) Correlations of t-statistics of genes based on promoter DNA methylation and H3K27ac or H3K36me3 signals. X axis: tissues for promoter DNA methylation. Y axis: tissues for H3K27ac and H3K36me3 signals. “*” denotes significant positive correlations after FDR correction (FDR < 0.001). [file 12915_2020_792_MOESM2_ESM.tif]

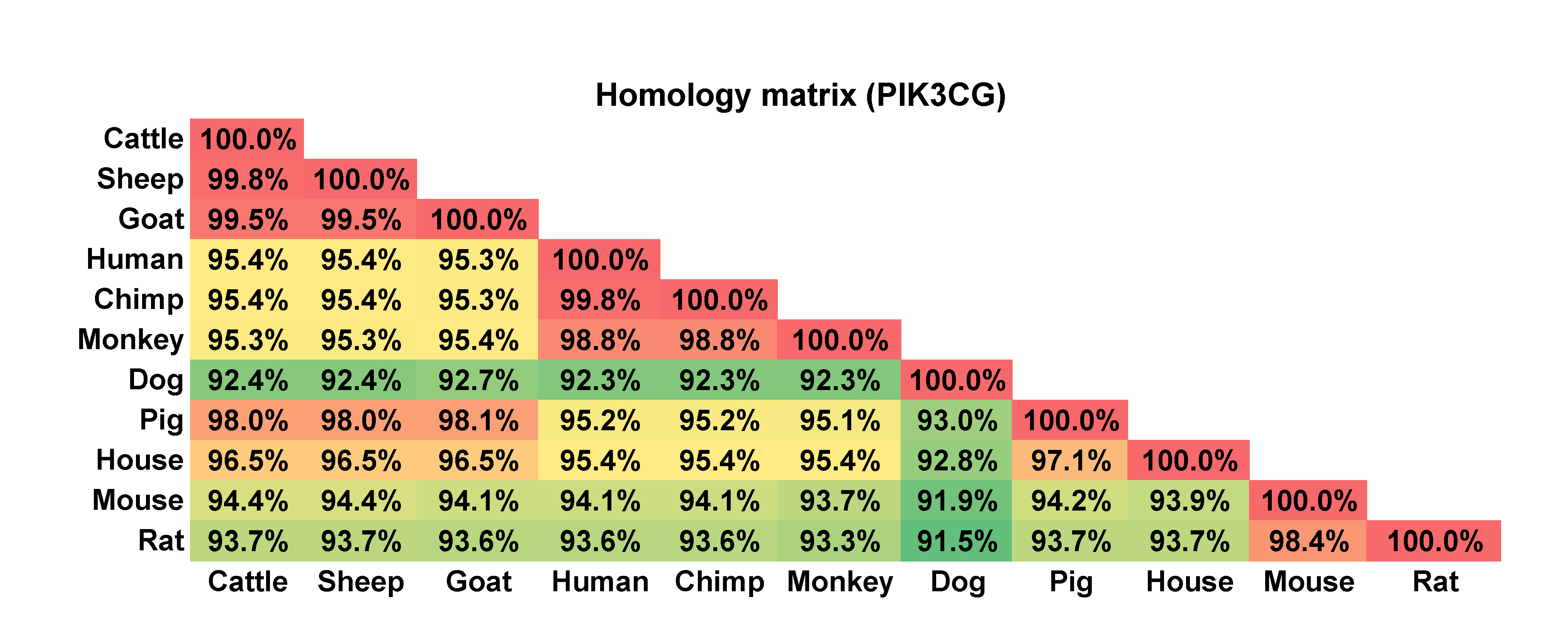

Supplement: Supplementary file 4 — Additional file 4: Figure S3. Alignment of the protein sequences of PIK3CG across different species. [file 12915_2020_792_MOESM4_ESM.tiff]

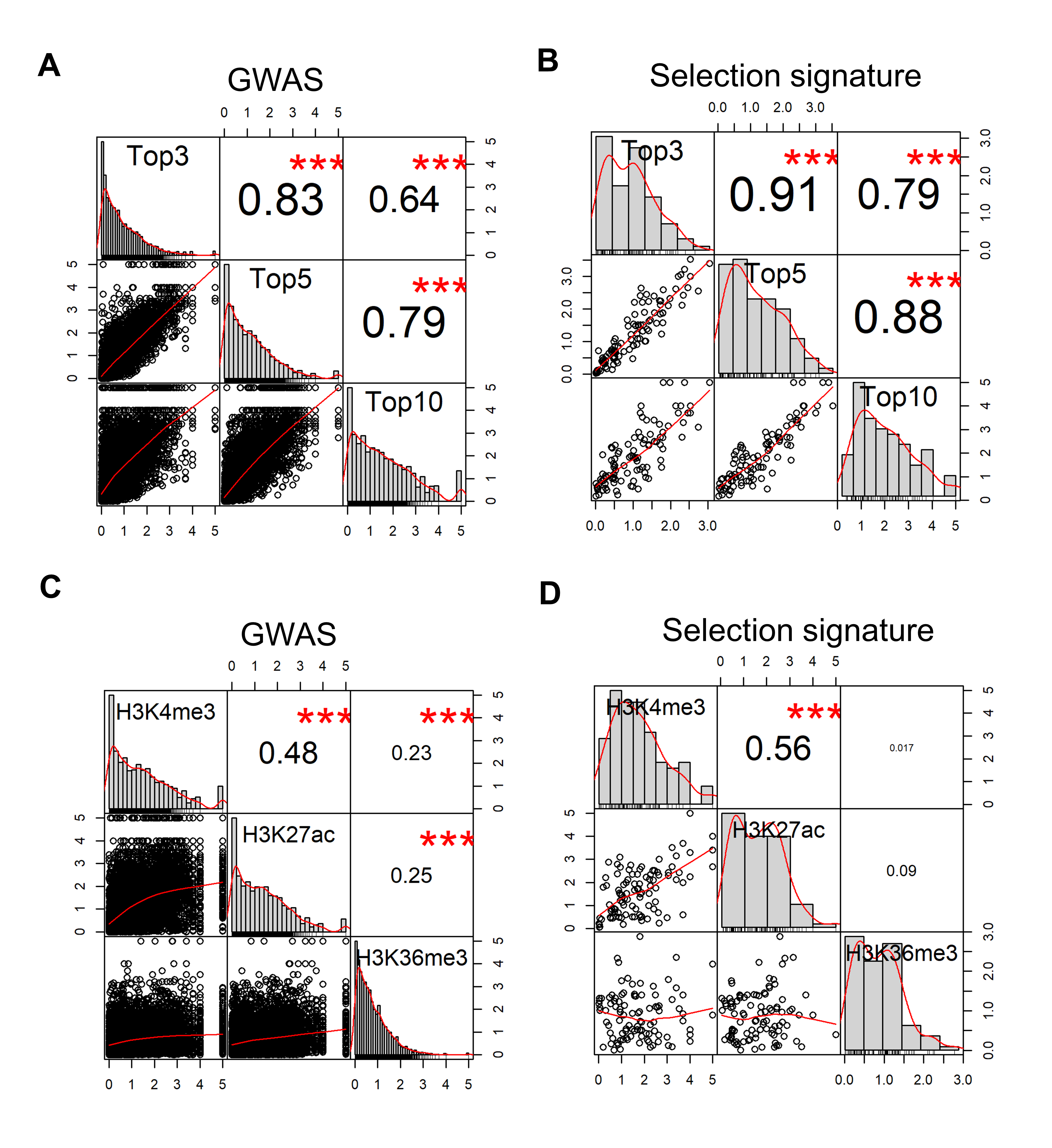

Supplement: Supplementary file 6 — Additional file 6: Figure S4. Correlations of enrichments among three criteria and three histone marks. Correlations of enrichments for GWAS signals (A) and selection signature (B) among top 3%, top 5%, and top 10% genes with tissue-specific H3K4me3, respectively. Correlations of enrichments for GWAS signals (C) and selection signature (D) among three histone marks (top 5%). [file 12915_2020_792_MOESM6_ESM.tif]
